# A critical review of fibrous polyurethane-based vascular tissue engineering scaffolds

**DOI:** 10.1186/s13036-022-00286-9

**Published:** 2022-03-24

**Authors:** Sonia Fathi-Karkan, Behnaz Banimohamad-Shotorbani, Sepideh Saghati, Reza Rahbarghazi, Soodabeh Davaran

**Affiliations:** 1 Student Research Committee, Tabriz University of Medical Sciences, Tabriz, Iran; 2 Department of Medical Nanotechnology, Faculty of Advanced Medical Science, Tabriz University of Medical Sciences, Golgasht St, Tabriz, Iran; 3 Drug Applied Research Center, Tabriz University of Medical Sciences, Tabriz, Iran; 4 Department of Tissue Engineering, Faculty of Advanced Medical Sciences, Tabriz University of Medical Sciences, Tabriz, Iran; 5 Stem Cell Research Center, Tabriz University of Medical Sciences, Tabriz, Iran; 6 Department of Applied Cell Sciences, Faculty of Advanced Medical Sciences, Tabriz University of Medical Sciences, Tabriz, Iran

**Keywords:** Polyurethane-based scaffolds, Engineered vascular grafts, Tissue engineering modalities, Angiogenesis

## Abstract

Certain polymeric materials such as polyurethanes (PUs) are the most prevalent class of used biomaterials in regenerative medicine and have been widely explored as vascular substitutes in several animal models. It is thought that PU-based biomaterials possess suitable hemo-compatibility with comparable performance related to the normal blood vessels. Despite these advantages, the possibility of thrombus formation and restenosis limits their application as artificial functional vessels. In this regard, various surface modification approaches have been developed to enhance both hemo-compatibility and prolong patency. While critically reviewing the recent advances in vascular tissue engineering, mainly PU grafts, this paper summarizes the application of preferred cell sources to vascular regeneration, physicochemical properties, and some possible degradation mechanisms of PU to provide a more extensive perspective for future research.

## Introduction

The occurrence of vasculitis and atherosclerosis can lead to the promotion of ischemic changes in the target organs. When vascular occlusion and pathological remodeling are extensive, it is necessary to use vascular grafts to circumvent the ischemic conditions. In most circumstances, vascular autografts, allografts, and xenografts exhibit poor physiological function associated with autoimmune response, several surgical procedures, and the risk of infection. Due to donor shortage and the above-mentioned issues, the advent and development of de novo strategies for the synthesis of engineered vascular grafts are mandatory [1]. As a correlate, studies targeting semi-synthetic and synthetic vascular grafts have needed to be increased during recent years. To develop an engineered vascular unit, the application of certain components and biomaterials comparable to in-vivo ECM properties is recommended.

In vascular tissue engineering, certain types of cells and biomaterials with specific physicochemical properties are used. The engineered structures should possess features to tolerate continuous physiological stresses while do not promote immunological response, inflammation, and thrombosis [2]. In addition, to mimic natural vessel structure, the luminal surface of the scaffold should support cell adhesion, flattening, migration, proliferation, and functional maturation [3]. Along with the application of specific scaffolds for vascular tissue engineering, the selection of appropriate cell lineage is helpful in structural stability and in-vivo integration. To this end, various autologous, allogenic, and xenogeneic cell types in mature and progenitor states (stem cells, and iPSCs) have been examined in numerous experiments [4]. Biomaterials used for engineered vessels should be easily malleable and flexible enough to provide a three-dimensional (3D) surface for cell attachment and phenotype acquisition. After cell attachment to the luminal surface, the functional cells are stimulated to remodel their ECM via traction forces, and proteolytic activity. An appropriate milieu allows the cells to efficiently sprout and form a tube-like structure [5]. Biodegradable biomaterials are appropriate candidates for optimizing therapeutic equipment, such as temporary prosthetics 3D porous structures, tissue engineering scaffolds, and controlled drug release systems [6].

Due to direct mechanical pressure and the presence of several enzymes inside the circulation system, the use of slow-rate degradable scaffolds or prosthetics is mandatory in vascular engineered structures. In this regard, polyurethane (PU) was introduced as one of the most critical synthetic polymers for implantable vascular grafts. Several studies have shown that PU possesses appropriate biocompatibility, and prominent mechanical properties such as high tensile strength, toughness, and resistance to degradation [3]. This review article aimed to highlight the application of PU-based structure for vascular tissue engineering prepared with different modalities.

## PU: chemical structure and properties

PUs are a family of multiblock copolymers containing a carbamate (urethane) group in their backbones [7]. These polymers are polymerized through step-growth polymerization, in the presence of a catalyst by reacting at least two isocyanates (hard segment) functional groups with hydroxyl or alcohol groups (soft segment). The soft segment is usually polyester, polyether, or poly alkyl diol with a molecular weight ranging between 500 and 5000 kD. The hard segment is usually an aromatic diisocyanate that reacts with a low molecular weight diol or diamine which is called a chain extender to form the urethane’s aromatic oligomer urethane-urea with a molecular weight of 300 to 3000 kD. Thus, reaction urethane linkage was created by reacting an isocyanate group (—N=C=O) with alcohol or hydroxyl (—OH) group. The final product is a polymer containing the urethane linkage, −RNHCOOR’-. As shown below, the urethane linkage is created by the reaction of isocyanate with alcohol or hydroxyl groups. In the molecular structure, the existence of short-chain diol [OH—R’—OH] helps to extend the chain [8].

$$ \mathbf{R}-\mathbf{NCO}+{\mathbf{R}}^{\prime }-\mathbf{OH}\mathbf{\to}\mathbf{R}-\mathbf{NH}-\mathbf{COO}-{\mathbf{R}}^{\prime } $$

**Fig. 1:**
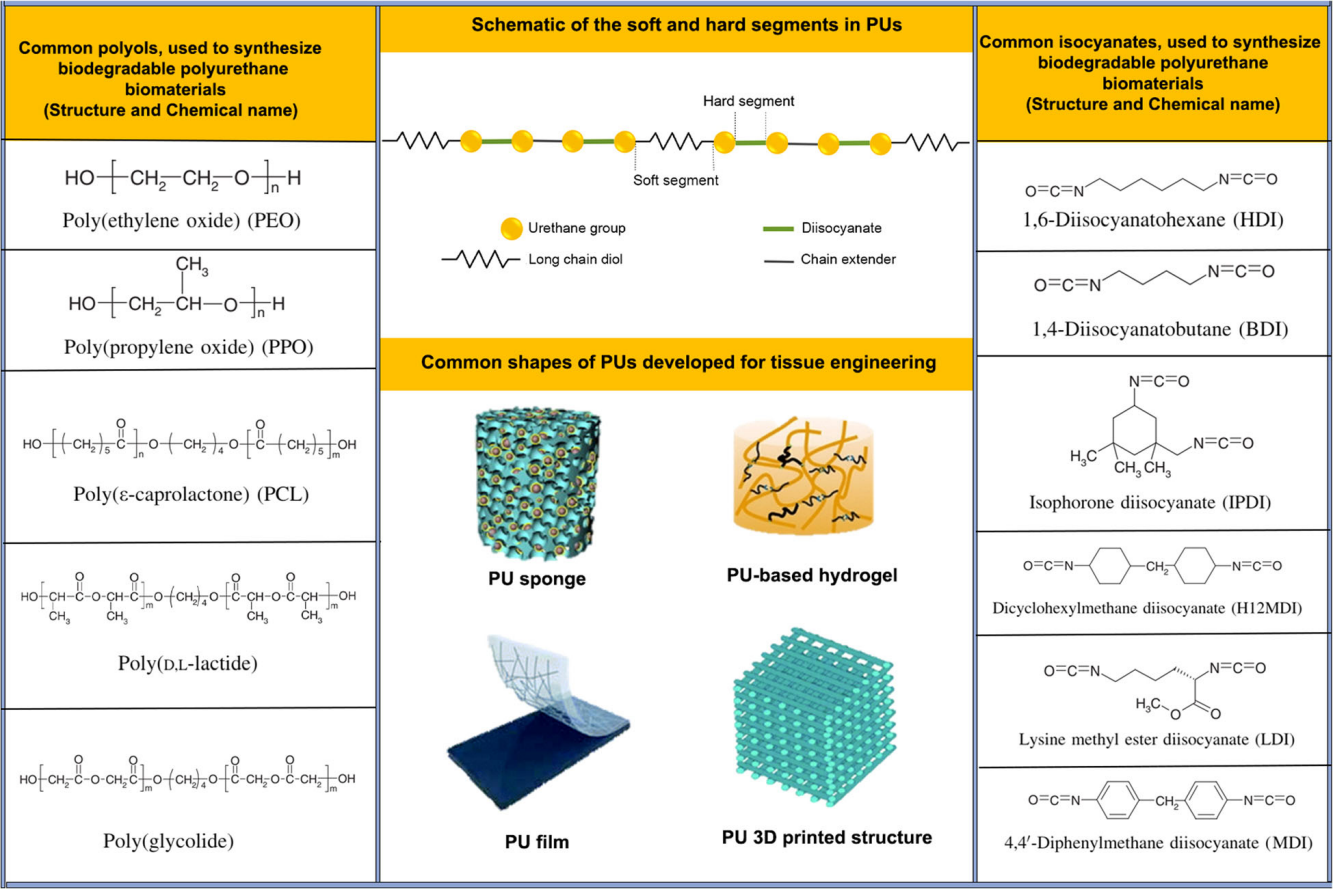
Schematic of the soft and hard segments in PUs and the formation of hydrogen bonds in the hard segments

Physicochemical properties of PU’s can be modified based on hard and soft segments components’ chemistry and molecular weight. Every change in molecular weight and molar ratios can lead to a wide variety of physical properties [hard, brittle, or soft materials]. The flexibility of the final component is associated with the quality and percent of the soft segment while the hard segment is the main factor affecting the structure strength. The choice of hard, soft, and chain extender segments can create materials with different mechanical properties, making PU an attractive biomaterial for engineered structures [9]. Besides, excellent mechanical properties, acceptable biocompatibility, hemo-compatibility, hydrolytic, and oxidative PU resistance make it convenient vascular scaffolding [10]. Researchers have commonly used PU as the main scaffolding substrate for subsequent logic as follows; Selection is mainly focused on the application of different values of the hard segment of hard segments (isocyanate), a chain extender (alcohol or amine), and soft segments (polyol). In case, alcohol is used as an extender chain, PU is synthesized while the addition of an amine group can help us to produce PU-Urea (PUU). To increase the flexibility of the final component, the soft segment can be used. This segment can form amorphous chains. By contrast, the increase of hard segment ratio forms crystalline-like properties for the final scaffold. It is suggested that Urethane or urea bonds can change the strength of the scaffold. Owing to the high density of hydrogen bonds in this area, high glass transition temperature, and thermal stability is generated [11] (Fig. 1).

By selecting the appropriate type and different weight ratios of the hard and soft segments, it is possible to synthesize an extensive family of particular types of PU with unique characteristics. For example, Bionate® and Elast-Eon® are widely used for long-term implantable medical applications. All family members are made of the same materials, and the only difference is the weight ratio of soft and hard segments [12, 13]. In recent years, surface modification and improvement in PU-based scaffolds have received much attention. Modifications such as coating, and cross-linking with biocompatible polymers, chemical modification with biocompatible functional groups, or stabilization of biomolecules or plasma treatment are at the center of attention [14].

## Biomaterials for vascular tissue engineering

The possibility of repairing or replacing damaged tissue has always been the primary concern. Tissue engineering tries to design and construct functional and alive components after transplantation. Application of specific cell lineages and biodegradable scaffolds along with certain growth factors helps vascular regeneration. Similar to other scaffold types, in vascular engineering, the scaffolds should possess a certain biodegradability rate that allows the formation of natural ECM [15]. Vascular grafts using autologous vessels such as SV, radial artery, or ITA are the conventional modalities for replacing vessels and bypass purposes. In many cases, an autologous vascular graft is not possible due to donor shortage, low quality, infection, and donor site morbidity [16].

Considering these limitations, extensive experiments and studies in the field of TEBV have been done [17]. Synthetic materials such as PTFE, PET (Dacron), PVC, PA (Nylon), PP, PTFE, PET-based grafts, and polyesters, including poly(−caprolactone) (PCL), polylactic acid (PLA), and polyglycolic acid, have been successful in large-caliber vessels substitute such as in aortoiliac replacement and medium-caliber arteries around 6–8 mm [18, 19]. Unfortunately, most of these grafts cannot replace small-caliber vessels less than 6 mm such as coronary, infra-inguinal, and infrageniculate arteries because of high thrombotic rate, occlusion, inflammation, hyperplastic complications, and reduced patency rates [20]. Weinberg and Bell prepared the first multilayer TEBV that mimicked all three arterial histologic layers. They seeded bovine aortic ECs, SMCs, and fibroblasts on a collagen-coated Dacron mesh scaffold [21]. The medial layer formed with a mixture of collagen, and SMCs, and then covered with adventitial fibroblasts in Dacron mesh as mechanical support. The ECs were plated in the lumen. Encapsulation of fibroblasts inside collagen hydrogel exhibited an appropriate contraction capacity. This feature permits the contraction around a cast. Therefore, forming a tubular construct with a reduced length, but preserving internal diameter, and considerably increased strength is possible. ECs generated single endothelial lining with a large-molecule permeability barrier with the ability to express vWF and prostacyclin. The burst strength in novel TEBV was achieved around 120–180 mmHg compared to physiologic burst strength 2000 mmHg and 3000 mmHg in the human SV and internal mammary artery, respectively [22]. Koike et al. have formed functional and stable long-term blood vessels in mice for 1 year using co-implantation of HUVECs and MSCs without genetic manipulation. The cells were seeded in a 3D type I collagen-fibronectin gel. The existence of heterotypic interaction between MSCs and ECs committed MSCs into mural-like cells. Based on released data, this system is eligible for the examination of several factors on neo-vascularization, vasculogenesis, and vessel maturation in in in-vitro conditions which applies to the in-vivo milieu*.* Transplantation of EGFP-tagged HUVECs in a 3D structure to the mouse model showed these cells successfully integrated into the vascular structure [23]. In another study, Mi and co-workers designed a biomimetic construct for vascular tissue engineering. They used silk as tunica intima, PAM hydrogel as tunica media, and electrospun TPU as the tunica adventitia and assembled in a TLVG. The developed TLVG structure had suitable mechanical property and suture preservation strength for surgical implantation. Although they successfully constructed triple-layered vessels, leakage of liquid under pressure, characteristics of the “toe-region,” and high elasticity were challenging to achieve [24]. Nottelet et al. designed a cell-free PCL-based vascular graft with an internal diameter of 2 or 4 mm. The scaffolds were implanted to Sprague-Dawley rats in substitution of an infrarenal abdominal aorta portion to estimate the patency and resistance of implanted scaffolds’ in vivo over 12 weeks. They observed good surgical handling and suture retention properties and an almost complete endothelial coverage to the endoluminal graft surface after 6 weeks of implantation, but some intima hyperplasia formation was observed in all grafts after 12 weeks [25]. Some reports examined non-FDA-approved materials like PTMC as vascular tissue engineering scaffolds. PTMC (Mn = 437 kDa) was crosslinked by gamma radiation under nitrogen. This material was fabricated into tubes with an inner diameter of 3.0 mm and a wall thickness of 0.7 to 0.8 mm (similar to the size of the human coronary arteries at an average outer diameter of 3.54 ± 0.51 mm and average wall thickness of 0.89 ± 0.21 mm). These dimensional compatibilities permitted easy anastomosis of the vascular graft with the native blood vessels. The suture retention strength was in the range of 1.64 to 1.94 N mm^− 1^, compared to that of native ovine and porcine arteries, which were 8.9 and 10.8 N mm^− 1^, respectively. The tubular PTMC was measured with a radial (or circumferential) tensile strength of about 1.6–1.9 MPa and elongation at 200–350% break. These mechanical properties were very similar to human arteries and could be further adjusted by varying the structure porosity and pore sizes [26].

## Nanofibers in tissue engineering

**Fig. 2:**
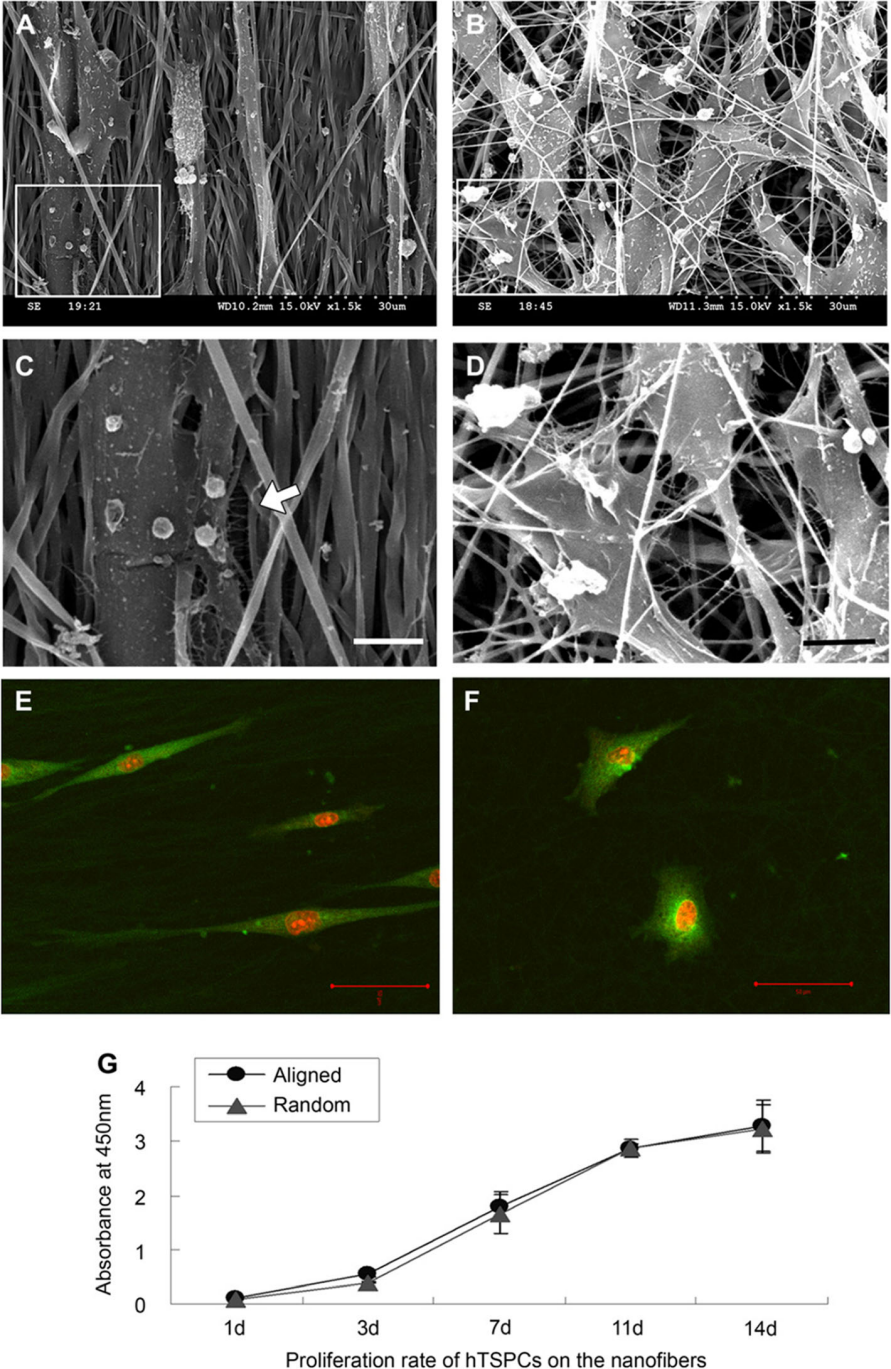
Morphological changes of tendon stem cells growing on the scaffolds. (**A**) and (**B**) show cultured cells on the aligned and randomly-oriented scaffold, respectively. (**C**) and (**D**) High magnification of selected areas in (**A**) and (**B**) show the cell-matrix adhesion between cells and nanofibers. Arrow in (**C**) shows a filament-like structure on the aligned scaffold. (**E**) and (**F**) Confocal micrograph of CFDA-stained elongated tendon stem cells on the aligned and randomly-oriented scaffold, respectively. (**G**) Cell proliferation on the aligned and randomly-oriented scaffolds. Copyright 2010 Elsevier

Imitation of the ECM structure is one of the main challenges of tissue engineering. Among the existing approaches to fabricate artificial ECM, nanofibers have shown the most promising results [27]. One of the most promising methods for manufacturing nanofibrous vascular grafts is electrospinning, making us able to fabricate aligned or random nanofibers with a high surface-to-mass ratio [17]. These scaffolds are applicable in tissue engineering, wound healing, sensor development, and controlled release of therapeutic agents [28]. Nanofibers could mimic and resemble the native environment and the ECM of cells virtually. Contact of nanofibers with plated cells affects their morphology and organization of the cytoskeleton and modulates stress transition into the cell [14]. It was reported that the concentration gradient of immobilized peptides can improve the proliferation, differentiation, and mineralization of plated cells [29]. In other studies, it was shown that the gradients of pore size on starch-PCL blend scaffold enhanced the efficiency of cell seeding and cell distribution rate [30]. It is thought that aligned nanofibrous are more effective on the migration and differentiation of plated cells in-vitro and in-vivo conditions compared to random nanofibrous structures (Fig. 2) [31].

### Nanofibers in cardiovascular tissue engineering

The development of nano- and micro-scale fibers is possible using various materials like polymers, ceramics, and even their composites. Polymer-based vascular electrospun scaffolds such as PU, PLA, and PCL are promising paths to answer the increasing demand for vascular replacements [32]. PLGA/type I collagen/elastin nanofibers could be fabricated on a circular mandrel as a vascular graft [33]. Besides, Hybrid or multiple-layered vascular grafts can be fabricated using dual-electrospinning or by different electrospinning materials [34]. It has been shown that nano-topographically alignment of nanofibers and micro-patterned stripe substrates not only helps mechanical compliance of vascular graft [35], but also regulated the behavior of ECs [36] such as cell distribution, migration velocity [37], and morphogenesis [38]. Yu et al. constructed a small-diameter vascular scaffold fabricated using TPU and silk fibroin. The scaffold was composed of an inner aligned layer and an outer random layer of nanofibers. Compared to human coronary arteries, adhesion, migration of cells, and good mechanical properties were comparable [39].

## Applications of PU-based scaffolds

PU is considered one of the appropriate blood-compatible and biocompatible materials with many applications as adhesives, rigid foams, elastomers, resins, and coatings [40]. The synthesis of the first biomedical grade polyether PU showed suitable mechanical properties, including resistance to flex-fatigue, high elasticity modulus, and good stability over long implantations [41]. Unfortunately, it has been reported that PU has low cell interaction and bio-stability in the long-term in in-vivo conditions [42]. In temperatures around 10–20 °C below the melting point of the medical-grade TPU, the hard block morphology continues to evolve to produce a reversible improvement in shear viscosity and elasticity of the TPU by increasing the time of synthesis. Degradation and cross-linking reactions happen at above melting temperature and lead to permanent enhancement of the shear viscosity and the elasticity of the TPU [43]. In artificial vascular engineering, the minimum amount of elongation break and the tensile strength have been reported around 40% and 1.4 MPa, respectively. Interestingly, PU nanofibers’ tensile strength up to 10.56 ± 2.12 MPa was detectable using coated polyaniline [32]. Analyses have revealed no toxicity effect of tubular PU on MDSCs [44].

## PU-based vascular scaffolds

Injured vessels can be replaced by artificial substrates to reduce, not completely but in part, the rate of deformation, looseness, and destruction [45]. Ideal artificial vessels should be resistant to corrosion, degradation, and mechanical fatigue and possess suitable porosity to exchange nutrients with supporting cell proliferation. Besides, the transplanted structure should be adaptable to match between artificial and autologous vessels. To avoid thrombus formation, hemo-compatibility will decrease the possibility of clot and thrombosis with time-dependent biodegradation (Table 1) [60]. It has been indicated that high ECs adhesion and fast endothelialization of PU, PCL, and PLGA-based scaffolds make them a suitable candidate for engineered vascular scaffolds. High flexibility, tear-resistance, cell affinity, biocompatibility, the possibility of chemical modification with proteins, the similarity in mechanical properties of PU make it a suited candidate for blood bags, catheters, blood vessel tissue engineering, wound dressings, and artificial heart valves [61]*.* Besides good processibility and other advantages of PU, the low hydrophilicity of PU is an issue that can be improved by some approaches such as surface modification or blending [32]. PU-based vascular scaffolds can be synthesized by several methods such as electrospinning, 3D printing. Their surfaces can be modified by coating to promote cell adhesion and proliferation rate. Also, PU-based vascular scaffolds can be fabricated in pure or multi-component and single-, double-, and triple-layered forms with other polymers or biomolecules [62]. These structures can be reached by different methods to mimic the native vessel microenvironment. The tubular porous triple-layered structure can be produced using the self-assembly method or by the combination of decellularized bovine aortas and synthetic polymers. However, these modalities are expensive and time-consuming. Synthetic polymers and natural polymers such as collagen, silk protein, and gelatin can be used for vessel engineering due to their excellent cell affinity and biocompatibility [34]. Other methods like gel spinning and lyophilization were also used for bi-layered silk protein vascular grafts with a porous outer layer and a fibrous inner layer [63]. It was suggested that a triple-layered vascular scaffold fabrication can be performed using electrospinning and the TIPS. Vascular grafts produced by TIPS have a low mass-to-volume ratio leading to poor mechanical attributes. Therefore, researchers increased the mechanical properties of electrospun fibers with the high interconnected porosity of TIPS, allowing an appropriate cell penetration. This method can easily be applied to vascular scaffold fabrication via an annular cylinder mold with a mandrel. The final triple-layered structure consists of electrospun TPU, TIPS TPU, and PPC indicated high mechanical properties, suitable dimensions, and high EC viability [34].

**Table 1:** Main elements influencing PU grafts in cardiovascular diseases

Parameters	Notation	Quantitative ranges	SPUs	TPU-Fibrin	PU-PCL	References
Physicomechanical features	- Elasticity depends on the usage, having proper modulus (without being too stiff). For cardiac tissue, proper range tensile strength is 3–15 kPa, modulus 10–50 kPa, and strain of 22–90%	Young, s modulus (MPa)	1.91 ± 0.49	1.19 ± 0.31	4.57–7.29	[46–53]
		Max tensile strength (σf, MPa)	9.48 ± 1.27	1.61 ± 0.37	60.45 ± 8.01	
		Ultimate strain (εf) (%)	521 ± 23	166 ± 27.6	512.32	
		T_g_ (°C)	−34	–	−41.70- −44.91	
		Tm (°C)	47.8	–	63.5–60.9	
		MW g/mol)	35,867	1000–3500	42,500–5000	
Degradation	- Polyester Pus: (hydrolytically unstable) - Polyether-based Pus (relatively insensitive to hydrolysis but susceptible to oxidative degradation) - Polyether-based PUs showed more stability than PCU and polyester-based PUs - If cell growth is restricted by slow degraded PUs, combining with fast-degraded polymer is the solution - The presence of antioxidants could inhibit the oxidative biodegradation					[34, 47, 54–56]
Porosity	- Porosity must allow cell/tissue infiltration - not promote degradation - support cell attachment and growth					[57, 58]
Blood- compatibility	- Blood-contacting PUs such as vascular scaffolds decreasing of platelet and white blood cell activation is required.					[58, 59]

**Fig. 3:**
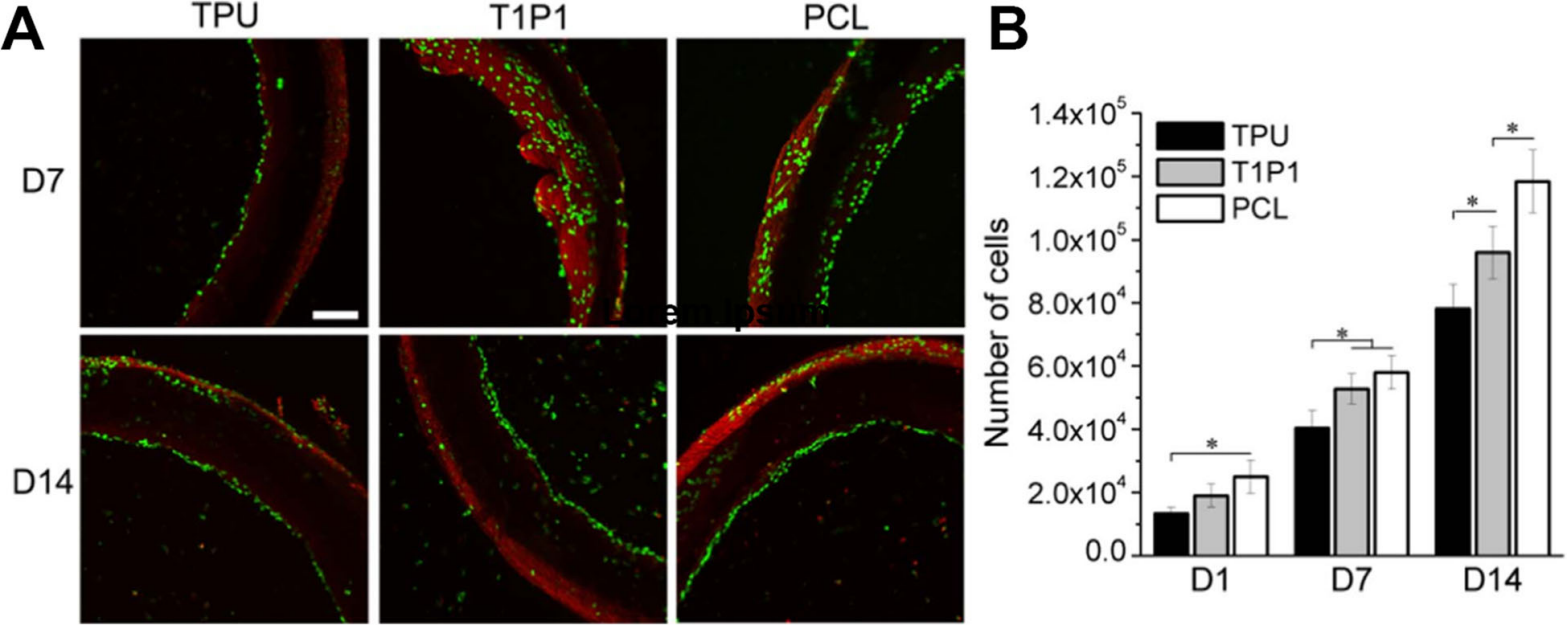
(**A**) Fluorescence photomicrographs of the Live/Dead assays of HUVECs cultured on TPU, T1P1, and PCL tubes for seven and 14 days. (**B**) Proliferation results of HUVECs cultured on the inner surface of open tubes for up to 14 days. Scale bar = 200 μm

It is thought that 3D printing is another way to obtain PU-based scaffolds. For this purpose, Esmaeili et al. used the extrusion technique, as additive manufacturing technology, with TPU and various concentrations of nanocrystalline hydroxyapatite nanopowder to manufacture artificial blood vessels. With increasing the content of ceramic additive, the young’s modulus and hardness value are intensified. The proper chemical stability and mechanical characteristics of final structures can be obtained by these methods [64]. Li et al., in 2019, developed biocompatible PANI-coated electrospun PU fiber scaffolds to improve hydrophilicity, adhesion, proliferation, migration, and differentiation of the HUVECs in in-vitro and in-vivo settings [32]. Previously, thermoplastic PU/PCL hybrids SDVS were fabricated using electrospinning and an assembled rotating collector. TPU/PCL hybrid SDVSs showed suitable mechanical and biocompatibility properties coated with HUVECs and mimicked elastin and collagen properties in blood vessels (Fig. 3) [65].

**Fig. 4:**
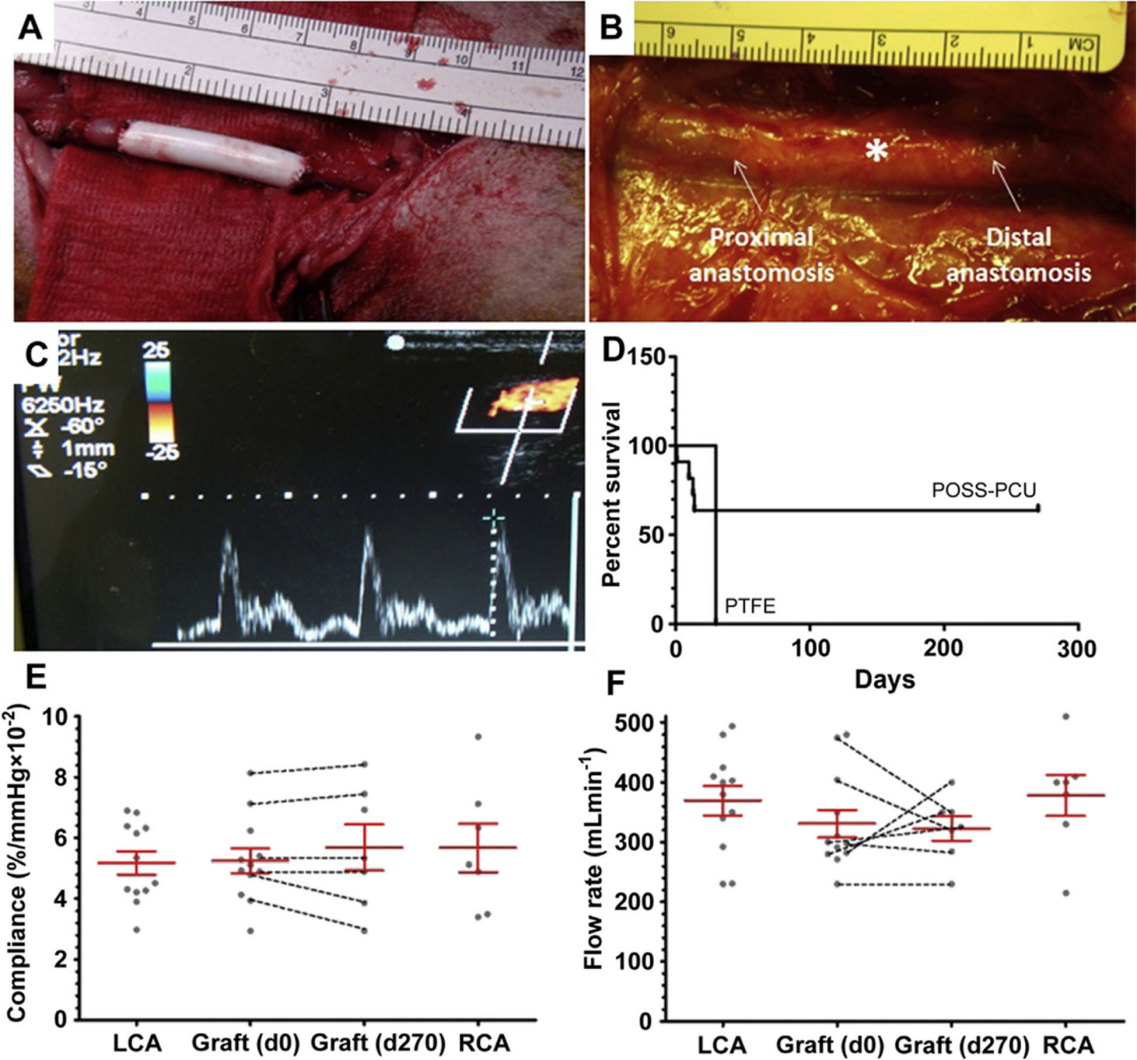
Images of a POSS-PCU vascular graft anastomosed end-to-end to the left carotid artery (**A**); Graft (marked *) after 9 months implantation surrounded by a thin layer of reddish fibrous tissue (**B**); Ultrasound image confirming flow through the graft (**C**); Kaplan-Meier survival curve of POSS-PCU and ePTFE vascular grafts implanted in the left carotid artery of adult sheep for 9 months (**D**). Compliance (**E**) and blood flow (**F**) rates through the intact left carotid artery, the graft just after implantation at day 0 (d0) and after 9 months before explantation (d270), and the right carotid artery. No significant differences were detected between the mean values for compliance (p ¼ 0.9706, n ¼ 12; n ¼ 7 for grafts at 9 months and right carotid artery. The change in compliance of the individual grafts further demonstrates no significant hardening or loss in the mechanical integrity of the grafts for the duration of the study. Similarly, mean flow rate (**F**) were unchanged (p ¼ 0.3693, n ¼ 12, n ¼ 7 for grafts at 9 months and right carotid artery across the groups, and while there were slight changes in the flow rates of the individual grafts, there was no significant change in blood flow levels compared to the contralateral the right carotid artery control

In another study, Li and co-workers fabricated heparin-grafted electrospinning PCU artificial vascular scaffolds. The final structure displayed excellent biocompatibility in in-vitro and in-vivo studies and with a low-rate thrombus formation [60]. Vascular EC adhesion and proliferation were improved by appending small quantities of GO and PEI into TPU and PCL backbones [66]. Some earlier studies indicated suitable mechanical properties of PCU vascular grafts with the elasticity between the artery and graft [67]. In 2014, PU-based artificial vessel grafts composed of polycarbonate and POSS were produced and implanted in the carotid artery in an ovine model. After transplantation, grafts did not show calcification, hyperplasia, and aneurysmal dilation and were comparable to native arteries in functional properties (Fig. 4) [68]. The parameters such as biostability and biocompatibility of PU-based scaffolds could be done by incorporating some antioxidants in the optimal amount [69], cross-linking with the corresponding agents (for example, elastin [70], glutaraldehyde [71], and N, N′-Methylenebisacrylamide [72]), the introduction of chain extenders, is essential to enhance the stability of electrospun PU scaffolds [73], co-electrospinning or combining PU with natural proteins (such as fibrinogen, bovine serum albumin [74], collagen [74], and gelatin [74]), and increasing hydrophilicity (using some polymers such as PEG [75]).

## Cell source

Along with several parameters affecting the quality of engineered vascular graft, the type of cell plated on the luminal surface of vascular grafts should also be monitored. It has been demonstrated that transplanted cells may face a low interaction rate with scaffold and native tissue and low levels of nutrients and oxygen can be reached to plated cells. Therefore, preconditioning such as hypoxic treatment and target differentiation of stem cells before transplantation looks beneficial. Due to the low levels of oxygen in cardiovascular diseases, transplanted stem cells will face hypoxia and exhibit apoptotic responses. Under the hypoxic condition, the expression of hypoxia-inducible factor-1 is elevated via engaging PI3K/AKT signaling pathway. This strategy will reduce the rate of cellular death and caspase-3 activation while enhancing angiogenesis response, and survival rate [76]. Post-treatment of MSCs with oxygen after transplantation could enhance the production of endothelial NOS and successful engraftment [77]. Results show that preconditioning and post-conditioning (before/after transplantation) could be beneficial in engineered vascular grafts [76].

### Autologous cell source

Due to the low-rate rejection of grafts, it is suggested that autologous cells are an interesting source. In several studies, autologous ECs, SMCs, and iPSCs of patients are the first choice for vascular tissue engineering [78]. Because iPSCs could be differentiated to all adult body cells, their differentiation capacity to SMCs [79], vascular mural cells, and ECs were reported [80]. In adult blood vessels, most of the cells are terminally differentiated and the main limitation in the application of differentiated cells is low proliferation potential [81]. Some differentiated cells such as adult human dermal fibroblasts [82], venous cells, autologous bone marrow cells [83], layers of myofibroblasts, mesothelium cells [84], and cell sheet of fibroblasts or SMCs with ECs [85] have been used clinically. Stem cells such as BM-MNCs have been widely used in vascular graft engineering. As a heterogeneous population, BM-MNCs include MSCs, mature ECs, late outgrowth EPCs, HSCs, monocytes, natural killer cells, B lymphocytes, and CD4^+^ and CD8^+^ T cells [86]. MSCs as adult adherent progenitor cells indicated the potential of self-renewal, rapid expansion [87], and can trans-differentiate into several lineages such as adipose, bone, cartilage, ligament, tendon, muscle, and cardiovascular phenotypes [88, 89]. The lack of MHC complex makes these cells possible to use as autologous or allogeneic cell sources [90].

Although MSCs could be isolated from several organs [91], there are some slight variations between different sources. Compared to bone marrow and umbilical cord, a higher frequency of MSCs could be gained from adipose tissue. Although the lowest harvest frequency was reported for umbilical cord MSCs, these cells exhibited showed the highest proliferation potential [92, 93].. The most common source of MSC is bone marrow for different approaches. The BMSCs could be obtained by removing non-adherent cells from BM-MNCs using Ficoll-Hypaque gradient centrifugation. About 0.01% of the mononuclear cells are MSCs and the differentiation potential will decrease by age [92]. Along with these tissues, skeletal muscle is another source for the isolation of MSCs which are so-called MDSCs. MDSCs are adhered cells and are isolated from digested skeletal muscle using enzymatic and mechanical digestion [94]. These cells can be cultured up to 250 passages without notable karyotyping changes [95]. In addition to MSCs, pericytes are capable of tissue regeneration [96] and express MSC-like markers. These cells can be used for regenerative purposes and are an alternative cell source for MSCs [97]. EPCs are another cell type that could differentiate into mature ECs. It has been indicated that these cells are eligible to furnish the luminal surface of engineered grafts [98].

### Allogeneic and xenogeneic cell source

Both ECs and SMCs could be used as autologous and allogeneic cell sources. Due to close contact with ECs and blood cells, rejection of graft is possible when allogenic cells are applied inside the vascular grafts [82]. Human extra-embryonic tissues include the umbilical cord, the placenta, and fetal membranes such as amnion and chorion [99]. Extra-embryonic MSCs could be isolated from amniotic fluid and umbilical cord [100]. As similar, WJ-MSCs are isolated from the sub-amnion, the inter-vascular zone, and the perivascular zone [101]. Compare to AM-MSCs, the proliferation rate, self-renewal potential, and anti-platelet adhesion of WJ-MSCs were higher. Also, the coagulation cascade is not activated by WJ-MSCs when plated in the luminal surface of vascular grafts [102]. Besides, the ease of accessibility, lack of substantial risks, preventing intact donor tissues from being sacrificed [103], the possibility of allogenic transplantation without ethical and immunological problems are the main advantages of WJ-MSCs [102]. ESCs are another cell source with the potential to produce all adult stem cell types which are restricted to particular lineages. Different studies have proved the successful differentiation of ESC into SMCs and ECs [104]. In ESCs, transfection strategies targeting hTERT can lead to the generation of immortalized ECs [105]. Acellular ECM was widely used as xenogeneic vascular scaffolds [106]. To date, there are no reports about the application of xenogeneic cell sources for vascular tissue engineering. Because of severe immune responses or zoonotic disease transmission, this strategy does not work appropriately.

## Cross-linking methods

At room temperature, network behavior of segmented PUs can be detected where challenging domains play the role of cross-links. Strained specimens tend to relax considerably, particularly at raised temperatures [107]. In chemical cross-linking, long triols or higher functionality polyol with or without long diol can be used in the soft segments and also short polyols (such as TMP combined with MDI) in the hard segments act as the chain extender. Compared to linear diols, short triols reduce the crystallinity of the hard segment and lead to decreased elastomer strength. PU elastomers display typical amorphous polymers’ typical mechanical behavior by using poly (oxyethylene) diol and TMP with hexamethylene diisocyanate. The application of tri- or multifunctional and linear polyols mixture affects differently. It is expected that chemical cross-linking superimposes the effect of physical cross-linking at a temperature lower than the melting point. At higher temperatures above the melting temperature, only chemical cross-links are operative. The improvement of solvent resistance can reduce tear strength and decrease stress-relaxation rate by random cross-linking of thermoplastic PU [107]. The type of the chain extender (diamine, diol) and its functionality (bi-, tri-functional) determine the degree of cross-linking and reactivity level of PURs, affecting mechanical and thermal attributes of final products [108]. The use of polar solvent solubilities such as dimethylsulfoxide or tetrahydrofuran is possible for those PURs whose cross-linking degree is deficient, making these PURs appropriate for use in emulsion freeze-drying modeling and electrospinning [109].

**Fig. 5:**
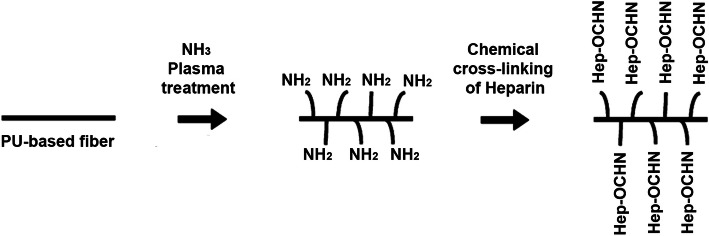
Schematic view of heparin grafting on PCU surface by plasma and chemical processing: PCU-NH2 (NH3 activated polyurethane blood vessels) and PCU-Hep combined with plasma treatment and chemical grafting method

**Fig. 6:**
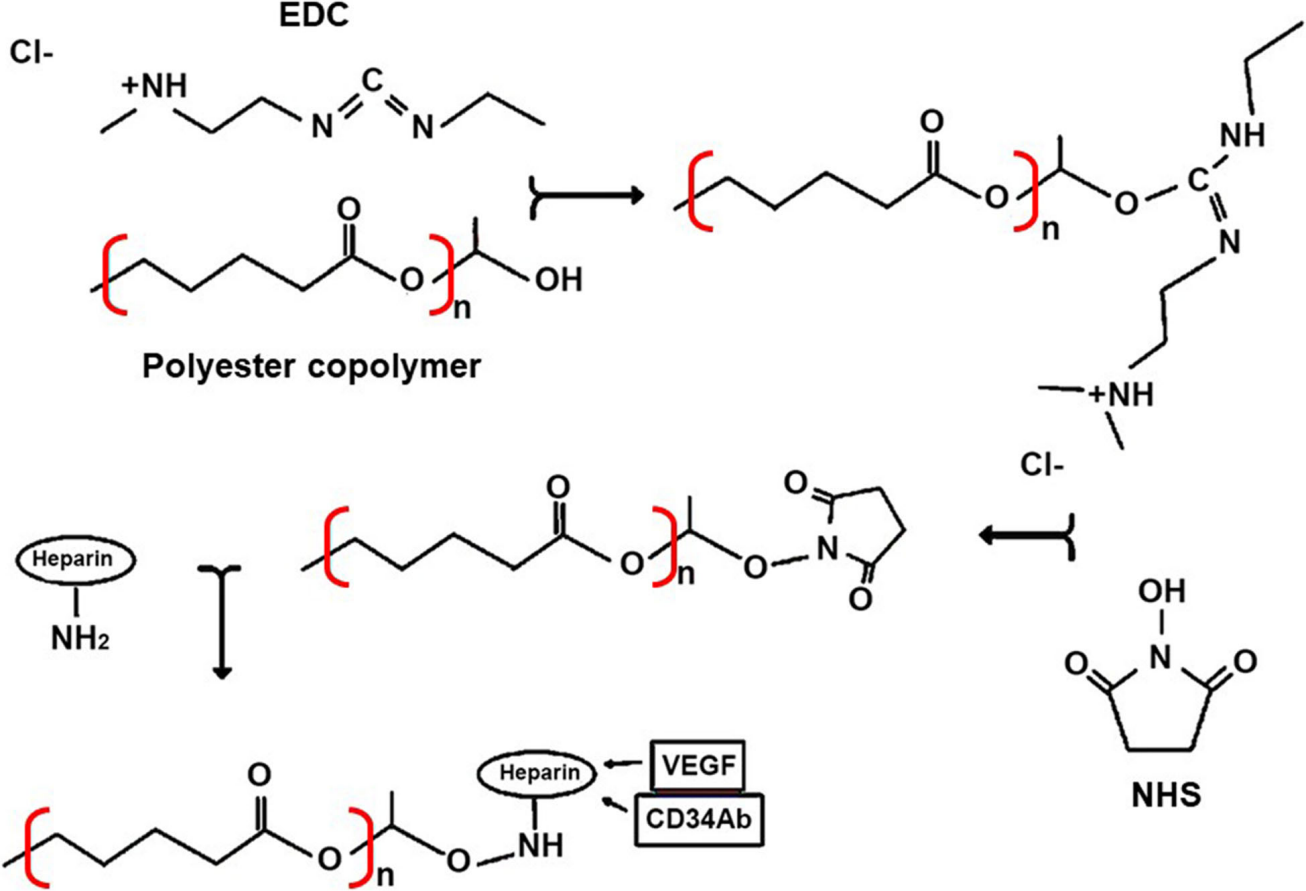
The applied reaction of EDC-NHS for the Heparin cross-linking via activating the hydroxyl functional group and binding the amine and then VEGF and antibody loading against CD34

To fabricate PCU vascular scaffolds, Li et al. used NH_3_ plasma treatment and chemical cross-linking of anticoagulant heparin sodium (Fig. 5). Their products showed excellent biocompatibility in in-vivo and in-vitro environments with low hemolysis and reduced intravascular thrombus. No significant improvement was observed in the density, porosity, mechanical properties [60]. Melchior et al. reported two coating approaches of heparin-cross-linked surfaces to load VEGF or immobilize anti-CD34Ab. EDC and NHS reaction was used for heparin cross-linking on the surface. Cross-linked heparin not only protects the bioactivity of bound proteins but also enhances the efficacy of VEGF delivery (Fig. 6) [110]. A combination of physical and chemical cross-linking using a long triol was done on the segmented PU. Data have indicated that the molecular weight of PU increases by lower concentrations of the triol, and cross-linked products are obtained at higher concentrations. Chemical cross-linking does not affect soft segment glass transition, tear strength, and hardness, but the tensile strength and strain at break can be enhanced by chemical cross-linking. The degree of cross-linking and elongation could affect the stress-relaxation at room temperature [107].

Safikhani et al. evaluated the effect of cross-linking reaction on a 3D bi-layered PU-gelatin electrospinning scaffold. Besides good viability and attachment of L929 fibroblast cells, a cross-linking reaction increases the ultimate tensile strength and reduces the modulus, swelling ratio, absorption, and hydrolytic biodegradation rate. These structures show primary mechanical and physicochemical attributes to support neo-tissue formation. Although the cross-linking delays the polymer degradation process, breaking down the macromolecules obtained from the cross-linking will increase the degradation ratio [111]. To fabricate a water-resistant scaffold, it is better to cross-link using glutaraldehyde [111]. Hao et al. fabricated LWPUs composed of PEG and PCL as the soft segments, and L-lysine, L-lysine ethyl ester diisocyanate, and 1, 3-propanediol as the hard segments. Final LWPUs showed appropriate stretchability, high strength, support cell adhesion, and proliferation of non-toxic degradation products. Protein adsorption and platelet adhesion were decreased due to the hydrophilic surfaces of LWPUs. The secretion of a pro-inflammation cytokine such as TNF-α is in low amounts while anti-inflammation cytokines such as IL-10 are in the highest amounts. The culture of adherent macrophages on the LWPU surface increased macrophage polarization into healing type (M2) [112]. The extra amounts of isocyanate groups (−NCO) produce carbamide after reaction with the water. Then carbamate and carbamide cross-linked to isocyanate and produce elastic cross-linking domains to improve the elasticity and tensile strength of LWPUs [112]. Those solutions with more hard segments and higher concentration lead to a higher level of entanglements and physical chain cross-linking [28].

## PU-protein interactions

Following implantation, cellular and protein interactions between the body and the biomaterial occur. The immune system participates in the biological integration of implants and biomaterials, such as foreign body reactions, wound healing, inflammation, and osseointegration [113]. Aortic intima includes some aligned micro-grooves at the direction of blood flow [114], so various studies have investigated the surface structure’s effect. The cell behavior such as differentiation [115], cell alignment [116], adhesion, and migration [117], not only is associated with scaffold composition but also depends on some physical features [114] such as surface topography [113] and architecture [118]. It was reported that PU with surface modification decreased platelet binding and activation [114]. Submicron textured PU surfaces remarkably can decrease bacterial cell adhesion under static and shear status [119]. NO produced by ECs on the surface of blood vessels takes part in the antibacterial, anti-inflammation, and anti-platelet activation/aggregation.

In this regard, Li-Chong et al. investigated PU with both NO release and surface texturing. Data indicated significant enhancement of the plasma coagulation time coincided with decreased platelet adhesion and activation. Besides, the risk of thrombosis and blood coagulation was reduced. This incorporated strategy showed a synergistic influence on decreasing bacterial adhesion of microorganisms, including *Staphylococcus aureus*, *Staphylococcus epidermidis*, and *Pseudomonas aeruginosa* [114]. Chemistry and some physicochemical properties such as topography, size, and shape can affect the immune system response. Designing immune-modulatory biomaterials and directing host immune responses to healing phenotypes should be considered [120]. The size of the particle, surface morphology, and adsorbent chemistry could control protein adsorption and the structural changes of adsorbed proteins on the hydrophilic surfaces in fewer than hydrophobic materials [121]. In 2018, Ruichao et al. investigated pore sizes of PU scaffolds on macrophages polarization. The smaller pore size of the WBPU scaffold could increase RAW 264.7 cell polarization to the M1 phenotype at the early stage, initiating a pro-inflammatory response. Recruiting more macrophages and polarization to the M2 phenotype after subcutaneous implantation of WBPU exhibits anti-inflammatory properties. Thirty days after implantation, filling up the internal pores reduced macrophage population and scaffold interaction, accelerating tissue repair [118]. In 2020, Sören et al. studied the relation of the roughness of PU surface, cell morphology [such as circularity, elongation, and cell spreading], and CD molecule expression of immune cells such as monocytes, NK cells, and T cells. Results showed no effect of surface roughness [113].

In another study, Morita et al. showed the relation between PU nanofibers’ diameter and protein adsorption, conformation, and activity. They supposed that hydrophobic interaction could cause protein adsorption and clustering of hydrophobic hard PU segments. Proteins such as bovine serum albumin and lysozymes were, but not on non-stretched surfaces, adsorbed on the stretched PU nanofibers. It is thought that protein adsorption on stretched PU nanofibers is proportionally enhanced with the reduction of diameter. The activity of adsorbed proteins on the nanofibers with diameter 950 nm (thick ones) was decreased because of massive conformational changes, while the thin nanofibers (480 nm diameter) maintains the natural shape of proteins and indicated a higher activity rate [121]. Antibacterial and antifouling properties of PU were reported by several studies [122]. It has been shown that covalent immobilization of chitosan and citric acid on PU-based materials could develop antibacterial and biocompatibility properties [123].

## The degradation rate of PU-based scaffolds

The rate of tissue growth and scaffold degradation should be coordinated. In-vivo biodegradation of PU is up to 30% of its transplanted mass during 6 months [124]. It is thought that this value is enough time for vascular cells proliferation and building up networks to maintain convenient mechanical support [34]. Environmental stress cracking delineates a failure of polyether PUs under strain is practical in in-vivo environmental elements including residual stress, oxidative processes, amount of ether in the soft segment, and foreign body response and related cells [40]. Oxidative degradation is considered the particular element that was leading PU cracking [125], and soft PUs show more amenability to oxidative degradation [126]. PUs with aromatic disuccinates indicated more bio-stability than aliphatic PUs that are amenable to surface cracking and oxidative degradation [127]. Increased stability of the PUs comprising aromatic disuccinates is understandable with their capacity to develop a hard segment in the crystal lattice due to molecular arrangement and strong intermolecular attraction through p-electron interaction. The growth of cells may be restricted by slow degradation of PU due to occupied space, incorporating the fast-degrading polymers such as PPC and an aliphatic polycarbonate composed of propylene epoxide with PUs is the solution [34]. Different kinds of urethane degradation include photo, thermal, ozonolytic, hydrolytic, chemical, enzymatic, and oxidative degradation [128]. Nevertheless, two primary degradation processes of polymers are hydrolysis and oxidation, leading to graft failure [129].

Photo degradation describes a photolytic degradation happening in the absence of oxygen, but most of the time, oxygen is present; therefore, oxidation is a usual photo degradation reaction [128]. There are some photo stability procedures, but this kind of degradation is not common in grafts, so more discussion is avoided. In thermal degradation, the usual absorption of infrared radiation leads to thermal degradation and oxidation reactions. By increasing the temperature, segmented PUs indicates a two-stage degradation procedure. Usually, the first stage happens at higher than 250 °C, and it is because of the thermolysis of urethane linkages and the second stage is because of the decomposition of the macrodiol component. A combination of thermal and mechanical effects could facilitate degradation [128], but this is not common inside the body. It is shown that PEsUs and PEtUs are highly resistant to ozone. Using ozone for sterilization for biomedical practice is usual. Ozone sterilization might lead to surface oxidation, hydrophilicity enhancement, and reduced contact angle [128]. Not only, do some organic chemicals such as organic acids, alcohols, esters, and ketones, cause chemical degradation of castable PU, but also high alkalinity or acidity could increase the rate of PU hydrolysis, but this is not usual in the body [130]. Calcification can happen in medical devices, particularly in PUs used for cardiovascular disease, and contributes to the loss of elasticity and limited functional lifetime. The high calcification capacity of PEtUs was previously reported [131]. The presence of metal ions such as Mg^2+^ and Fe^3+^ [132] and silk fibroin [133] could reduce the calcification rate. Ether-free, physically cross-linked, fully aliphatic, and polybutadiene-based PUU was resistant to calcification [134]. Enhanced calcification was reported in a higher PEG content in PEG/PCL-based PUs [124]. Regarding hydrolysis degradation, the hydrolysis resistance of PEtUs is better than PEsUs. Generally, most PUs (except PUs with polyester diols) show excellent hydrolysis resistance [135]. The higher temperature could improve water absorption [136], water solubility [137], and hydrolysis of Pus [138].

### Hydrolytic degradation of poly ester-urethane

**Fig. 7:**
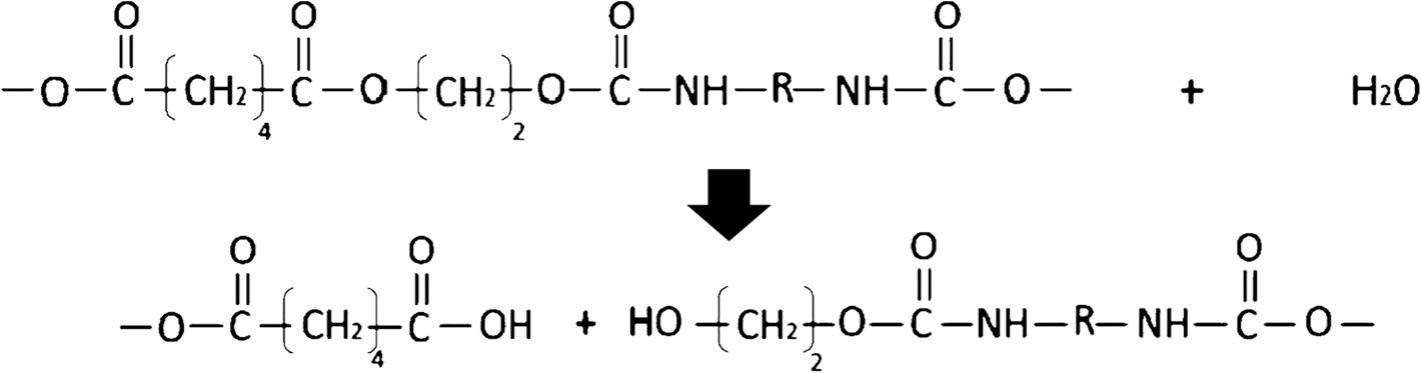
Schematic hydrolysis of poly ester-urethane which water reacts with a carboxylic ester link. The reaction breaks the polymer chain at the point of attack, producing two shorter chains. One of these chains ends in a hydroxyl group (−OH). The other ends in a carboxyl group (−CO_2_H) are acidic. This acidic carboxyl group speeds up the further hydrolysis of the polyester segments in the poly (ester-urethane), and the degradation becomes autocatalytic

The carboxylic ester link of the PEsU chain could react with water and break into two chains with hydroxylic (−OH) and acidic carboxylic (−COOH) end [139]. Hydrolysis could speed up by the carboxyl group and becomes autocatalytic [140]. Hydrolyzing of urea linkage gives two amines and carbon dioxide [141]. Additionally, hydrolyzing of urethane bonds could give amine, carbon dioxide, and alcohol [142] (Fig. 7). In the polymeric chains, the relative increase of methylene groups to other groups could improve the hydrophobicity of the derived PUs and makes them more resistant to hydrolysis [143]. A combination of ECM elements like chondroitin sulfate or hyaluronic acid into a PEA/4,4′-MDI/EG-based PU could improve the hydrophilicity and increase the tendency to hydrolytic degradation [139].

### Hydrolytic degradation of poly ether-urethane

Polyether-based components generally show high stability [144]. An in-vitro study of commercial PEtU (Elasthane™ 80A, DSM Biomedical) in deoxygenated PBS, neutral pH, at 37–85 °C indicated only backbone chain-scission of urethane (carbamate) links [145]. Ether components comparing polyester PUs show more resistance to hydrolytic degradation [146]. The moist and high temperature during autoclave sterilization leads to chain scission and tensile strength [147]. The existence of PEG in PU could increase hydrolytic degradation of poly (ester-ether-urethane). Water absorption reduces hydrolytic instability in bulk and leads to rapid cleavage of unstable ester bonds [148]. The lower content of PEG, higher PCL content, and lower molecular mass improve hydrolytic stability. Hydrolytic stability PUs generally depends on the hydrophobicity/hydrophilicity of macrodiols [128]. Against hydrolytic degradation, PU elastomers could be stabilized in several ways such as avoiding the use of polyester diols [11], increasing the size by crosslinking [149], introducing side chains [150], surface modification [151], using aromatic isocyanates [152], Organic [153] and inorganic stabilizers [154].

### Enzymatic degradation

Enzymatic degradation is very important in the biomedical usage of PUs. The chronic inflammatory response to PU implants produces esterases [155]. During PU degradation, cholesterol esterase is the most active enzyme in PU degradation [156]. Other proteases included in PU degradation include lipases, papain, and urease [157]. The soft segments’ hydrophilicity affects the stability of PU against enzymatic hydrolysis [158]. PU with hydrophilic PEG shows more susceptibility to enzymatic hydrolysis [159], but PUs with macrodiols with linear aliphatic structure showed more stability in enzyme solutions [159]. It has been reported that degradation of PUs (both enzymatic and hydrolytic degradation) relies on the type of diisocyanate and macrodiol. The degradation process is much more convenient for short-carbon chain macrodiols and aliphatic diisocyanate [141]. Polyether-based PUs showed more stability than PCU and polyester-based PUs in in-vitro hydrolytic degradation tests, but high stress, oxidative environments, and enzymatic attack lead to considerably faster degradation [54].

### Oxidative degradation

The early degradation process of PCUs and PEtUs is oxidation, and the effect of enzymes is minimum [160]. Releasing reactive oxygen species from adherent cells is related to in-vivo oxidative degradation [161]. On the PU implant, the interaction of hydrogen peroxide (released from inflammatory cells [162]) and metal of the conductor coil lead to metal ion oxidation [160]. Oxidative biodegradation could be accelerated by the H_2_O_2_/CoCl_2_ system and hydroxyl radical (^•^OH). PEtUs look inclined to oxidative degradation [160]. In-vitro and in-vivo results indicated that oxidation results in chemical changes of PEtUs such as urethane linkages [163]. Oxidative degradation can be inhibited possibly by using a long methylene chain of polyether for the soft segment (high CH_2_/O ratio) [131], modification with siloxane-based polymers [164], partial substitution of the polyether SS with PDMS (for PEtUs) [165], using some antioxidant stabilizers including Santowhite®, Irganox [166], Methacrol 2138F [167], vitamin E (α-tocopherol) [168]. Compared to vitamin E, Santowhite® is more effective due to its stable phenoxy radicals and its ability to end more than one chain (by a single vitamin E molecule) [168]. Also, some researchers reported good efficiency of biological antioxidants such as vitamin E and C (increasing stability and controlling nearby cells’ metabolic activity). However, the biocompatibility of PU was decreased by increasing the antioxidant concentration and leading to toxic products in oxidative degradation [168]. The substitution of the siloxane segment [169], using polycarbonate ones [170] with polyethers of PU makes it more stable to oxidative degradation. Antioxidant-based inhibitors (such as Irganox and Santowhite) could be used in biomedical usage [166]. Christenson et al. [55] showed that antioxidants could inhibit the oxidative biodegradation of both PCU and PEtUs electrospun nanofiber scaffolds. The PCU degradation rate was noticeably lower than PEtUs and was limited to a thin layer of the PCU surface.

### Mechanical degradation

Mechanical energy, high shear, and stress during processing or utilization [171] could result in ozone-induced reactions, autoxidation, rupturing of polymer bonds [172], crack formation [173], and macroalkyl radical production. The reaction of macroalkyl radical and oxygen form peroxy radicals. Then usual oxidative chain reactions occur [174]. The type of loadings, such as torsion or tension, and the highest loading level determine cracks’ orientation [175]. A low-viscosity fluid could decrease the fatigue resistance of PU elastomers [176]. Structural and chemical changes of PU elastomers could be happening by stress. It was reported that static, uniaxial stress could decrease the tendency of the PEtUs to calcify [131].

### PU-based vascular graft degradation

Polyester PUs such as Vascugraft by B. Braun Melsungen AG (Melsungen, Germany) was first used as vascular grafts; regardless, initial reports showed promising biocompatibility; the graft was subjected to surface chemical changes and eventually degraded in vivo, which seems to be due to the hydrolytic instability of the polyester polyol as soft segments [177]. The next generation of vascular grafts has developed Polyether-based PUs, such as Pulse-Tec vascular access grafts (Newtek vascular products, North Wales, UK); although these constructs were insusceptible to hydrolysis but prone to oxidative degradation [178]. Another vascular graft made with polyetherurethaneurea is Vectra (Thoratec Laboratories Corporation, Pleasanton, Calif), which received Food and Drug Administration (FDA) approval in 2000. In a clinical trial, 142 patients received either Vectra or ePTFE vascular grafts, and after 12 months, no difference was found in the patency or complication rates of the two grafts. However, it was reported that the PU graft elongated over time after implantation, and the incidence of the pseudointimal formation nearby anastomosis was relatively high than that in the ePTFE grafts [179]. Recent research has focused on developing polycarbonate-based PUs vascular grafts because they eliminate most ether/ester linkages; therefore, they are hydrolytically and oxidatively stable and are less susceptible to biodegradation. In this regard, the excellent stability of the poly (carbonate-urea) urethane graft was indicated both in-vitro and in-vivo. In an animal study, the graft was implanted in aortoiliac arteries in 4 dogs for 36 months, and no evidence of polymer degradation was found [180].

## Conclusion and future prospects

This review focused on discussing PU-based constructs introduced as a vascular graft to overcome challenges such as intima hyperplasia due to compliance mismatch observed in vascular tubes being used. Compliance could be optimized by handling the concentration of hard and soft segments. PUs-based grafts are preferred over e-PTFE due to adequate compliance and mechanical features close to the native vessel. Moreover, the surface of PU grafts can self-heal instantaneously behind being needle puncturing, thus, resulting in the tiniest plasma leakage after anastomosis, in contrast to e-PTFE. Nevertheless, early in-vivo trials failed due to insufficient bio-strength and hydrolytic/oxidative degradation. To improve PU-grafts bio-strength, replacement of macrodiols with carbonates has been offered, which has demonstrated enhanced resistance to biodegradation, and hence, could stay at the graft site for an extended period. Besides all vascular tissue graft advancements, designing and fabricating small diameter vessels useful in some diseases such as kidney disease, managing degradation, and regeneration rate simultaneously and more accurately, minimizing immune body responses and platelet activation are challenging issues. It is required to do more investigation and examine innovative ideas to achieve an ideal graft with the least problems.

## Data Availability

Not applicable.

## Abbreviations

3D: three-dimensional
AM-MSCs: Amniotic Membrane Derived Mesenchymal Stem Cells
BMSCs: Bone Marrow-Derived Mesenchymal Stem Cells
ECM: Extracellular Matrix
ECs: Endothelial Cells
EGFP: Enhanced Green Fluorescent Protein
EPCs: Endothelial Progenitor Cells
ESCs: Embryonic Stem Cells
GO: Graphene Oxide
HUVECs: Human Umbilical Vein Endothelial Cells
iPSCs: Induced Pluripotent Stem Cells
ITA: Internal Thoracic Artery
TEBV: Tissue-Engineered Blood Vessels
LWPUs: Light-Crosslinking of Waterborne Polyurethanes
MDI: 4, 4′-Diphenylmethane Diisocyanate
MDSCs: Muscle-Derived Mesenchymal Stem Cells
MHC: Major Histocompatibility Complex
MSCs: Mesenchymal Stem Cells
NO: Nitric Oxide
NOS: Nitric Oxide Synthases
PA: Polyamide (Nylon)
PAM: Polyacrylamide
PANI: Polyaniline
PCL: Poly ε-caprolactone
PCU: Polycarbonate Urethane
PEG: Polyethylene Glycol
PEI: Polyethyleneimine
PEsUs: Polyester Urethane
PEtUs: Poly Ether-Urethane
PET: Polyethylene Terephthalate
PLA: Polylactic Acid
PLGA: Poly (L-lactide-co-glycolide)
POSS: Polyhedral Oligomeric Silsesquioxane
PP: Polypropylene
PPC: Polypropylene Carbonate
PTFE: Polytetrafluoroethylene
PTMC: poly (trimethylene carbonate)
PU: Polyurethane
PUU: poly (urethane urea)
PVC: Polyvinyl Chloride
SDVSs: Small-Diameter Vascular Scaffolds
SPUs: Segmented polyurethanes
SMCs: Smooth Muscle Cells
SV: Saphenous Vein
TIPS: Thermally Induced Phase Separation
TLVG: Triple-Layered Vascular Graft
TMP: trimethylolpropane
TPU: Thermoplastic PU
vWF: von Willebrand factor
WBPU: waterborne biodegradable polyurethane
WJ-MSCs: Wharton’s Jelly-derived MSCs

## References

[1] Pina S, Ribeiro VP, Marques CF, Maia FR, Silva TH, Reis RL, Oliveira JM (2019). Scaffolding strategies for tissue engineering and regenerative medicine applications. Materials.

[2] Tabata Y (2009). Biomaterial technology for tissue engineering applications. J R Soc Interface..

[3] Karkan SF (2021). Electrospun polyurethane/poly (ɛ-caprolactone) nanofibers promoted the attachment and growth of human endothelial cells in static and dynamic culture conditions. Microvasc Res.

[4] Chan XY (2017). Human pluripotent stem cells to engineer blood vessels, in Engineering and Application of Pluripotent Stem Cells.

[5] Edgar LT (2014). Mechanical interaction of angiogenic microvessels with the extracellular matrix. J Biomech Eng.

[6] Fathi Karkan S. S. Davaran, and A. Akbarzadeh, *cisplatinReaction engineering of step growth polymerization-loaded superparamagnetic nanoparticles modified with PCL-PEG copolymers as a treatment of A549 lung cancer cells*. Nanomedicine Res J. 2019;4(4):209–19.

[7] Crescentini TM (2019). Mass spectrometry of polyurethanes. Polymer.

[8] Gupta SK, Kumar A. Reaction engineering of step growth polymerization. New York: Springer Science & Business Media; 2012.

[9] Sonnenschein MF, Lysenko Z, Brune DA, Wendt BL, Schrock AK (2005). Enhancing polyurethane properties via soft segment crystallization. Polymer.

[10] He W (2013). The preparation and performance of a new polyurethane vascular prosthesis. Cell Biochem Biophys.

[11] Prisacariu C (2011). Polyurethane elastomers: from morphology to mechanical aspects 2011.

[12] Kurtz S, Siskey R, Reitman M (2010). Accelerated aging, natural aging, and small punch testing of gamma-air sterilized polycarbonate urethane acetabular components. J Biomed Mater Res B Appl Biomater.

[13] Gunatillake PA, Martin DJ, Meijs GF, McCarthy SJ, Adhikari R (2003). Designing biostable polyurethane elastomers for biomedical implants. Aust J Chem.

[14] Amani H (2019). Controlling cell behavior through the design of biomaterial surfaces: a focus on surface modification techniques. Adv Mater Interfaces.

[15] Hutmacher, D.W., M. Sittinger, And M.V.J.T.i.B. Risbud, Scaffold-based tissue engineering: rationale for computer-aided design and solid free-form fabrication systems 2004. 22(7): p. 354–362. 10.1016/j.tibtech.2004.05.005 15245908

[16] Zheng H (2020). Deconstruction of heterogeneity of size-dependent exosome subpopulations from human urine by profiling N-glycoproteomics and phosphoproteomics simultaneously. Anal Chem.

[17] Karkan SF, Davaran S, Rahbarghazi R, Salehi R, Akbarzadeh A (2019). Electrospun nanofibers for the fabrication of engineered vascular grafts. J Biol Eng.

[18] Toong DWY (2020). Bioresorbable polymeric scaffold in cardiovascular applications. Int J Mol Sci.

[19] Ye H (2018). Polyester elastomers for soft tissue engineering. Chem Soc Rev.

[20] Maitz MF (2015). Applications of synthetic polymers in clinical medicine. Biosurface Biotribol.

[21] Weinberg CB, Bell E (1986). A blood vessel model constructed from collagen and cultured vascular cells. Science.

[22] Konig G (2009). Mechanical properties of completely autologous human tissue engineered blood vessels compared to human saphenous vein and mammary artery. Biomaterials.

[23] Koike N (2004). Creation of long-lasting blood vessels. Nature.

[24] Mi H-Y, Jiang Y, Jing X, Enriquez E, Li H, Li Q, Turng LS (2019). Fabrication of triple-layered vascular grafts composed of silk fibers, polyacrylamide hydrogel, and polyurethane nanofibers with biomimetic mechanical properties. Mater Sci Eng C.

[25] Nottelet B, Pektok E, Mandracchia D, Tille JC, Walpoth B, Gurny R, Möller M (2009). Factorial design optimization and in vivo feasibility of poly (ε-caprolactone)-micro-and nanofiber-based small diameter vascular grafts. J Biomed Mater Res Part A Off J Soc Biomater Jpn Soc Biomater Aust Soc Biomater Korean Soc Biomateri.

[26] Song Y (2010). Flexible and elastic porous poly (trimethylene carbonate) structures for use in vascular tissue engineering. Acta Biomater.

[27] Shitole AA (2019). Clopidogrel eluting electrospun polyurethane/polyethylene glycol thromboresistant, hemocompatible nanofibrous scaffolds. J Biomater Appl.

[28] Mi H-Y, Jing X, Jacques BR, Turng LS, Peng XF (2013). Characterization and properties of electrospun thermoplastic polyurethane blend fibers: effect of solution rheological properties on fiber formation. J Mater Res.

[29] Moore NM (2011). Synergistic enhancement of human bone marrow stromal cell proliferation and osteogenic differentiation on BMP-2-derived and RGD peptide concentration gradients. Acta Biomater.

[30] Sobral JM (2011). Three-dimensional plotted scaffolds with controlled pore size gradients: effect of scaffold geometry on mechanical performance and cell seeding efficiency. Acta Biomater.

[31] Yin Z, Chen X, Chen JL, Shen WL, Hieu Nguyen TM, Gao L, Ouyang HW (2010). The regulation of tendon stem cell differentiation by the alignment of nanofibers. Biomaterials.

[32] Li Y (2019). Blood-compatible polyaniline coated electrospun polyurethane fiber scaffolds for enhanced adhesion and proliferation of human umbilical vein endothelial cells. Fibers Polymers.

[33] Stitzel J, Liu J, Lee SJ, Komura M, Berry J, Soker S, Lim G, van Dyke M, Czerw R, Yoo JJ, Atala A (2006). Controlled fabrication of a biological vascular substitute. Biomaterials.

[34] Mi H-Y (2016). Approaches to fabricating multiple-layered vascular scaffolds using hybrid electrospinning and thermally induced phase separation methods. Ind Eng Chem Res.

[35] Wu H (2010). Electrospinning of small diameter 3-D nanofibrous tubular scaffolds with controllable nanofiber orientations for vascular grafts. J Mater Sci Mater Med.

[36] Xu C (2004). Aligned biodegradable nanofibrous structure: a potential scaffold for blood vessel engineering. Biomaterials.

[37] Ahmed M (2018). Geometric constraints of endothelial cell migration on electrospun fibres. Sci Rep.

[38] Moon JJ (2009). Micropatterning of poly (ethylene glycol) diacrylate hydrogels with biomolecules to regulate and guide endothelial morphogenesis. Tissue Eng A.

[39] Yu E (2016). Fabrication and characterization of electrospun thermoplastic polyurethane/fibroin small-diameter vascular grafts for vascular tissue engineering. Int Polym Process.

[40] Adipurnama I (2017). Surface modification and endothelialization of polyurethane for vascular tissue engineering applications: a review. Biomater Sci.

[41] Boretos JW, Pierce WS (1968). Segmented polyurethane: a polyether polymer. An initial evalution for biomedical applications. J Biomed Mater Res.

[42] Tondnevis F, Keshvari H, Mohandesi JA (2019). Physico-mechanical and in vitro characterization of electrically conductive electrospun nanofibers of poly urethane/single walled carbon nano tube by great endothelial cells adhesion for vascular tissue engineering. J Polym Res.

[43] Lu G (2003). Rheology and extrusion of medical-grade thermoplastic polyurethane. Polym Eng Sci.

[44] Takanari K (2017). Skeletal muscle derived stem cells microintegrated into a biodegradable elastomer for reconstruction of the abdominal wall. Biomaterials.

[45] Zhang L, Feng Y. Bibliometrics and visualization analysis of artificial blood vessel research. Curr Sci. 2014:816–22.

[46] Boffito M, Sartori S, Ciardelli G (2014). Polymeric scaffolds for cardiac tissue engineering: requirements and fabrication technologies. Polym Int.

[47] Zhang X, et al. Design of biodegradable polyurethanes and the interactions of the polymers and their degradation by-products within in vitro and in vivo environments, in Advances in polyurethane biomaterials. Amsterdam: Elsevier; 2016. p. 75–114.

[48] Davoudi P, Assadpour S, Derakhshan MA, Ai J, Solouk A, Ghanbari H (2017). Biomimetic modification of polyurethane-based nanofibrous vascular grafts: a promising approach towards stable endothelial lining. Mater Sci Eng C.

[49] Khodadoust M, Mohebbi-Kalhori D, Jirofti N (2018). Fabrication and characterization of electrospun bi-hybrid PU/PET scaffolds for small-diameter vascular grafts applications. Cardiovasc Eng Technol.

[50] Yu E (2018). Development of biomimetic thermoplastic polyurethane/fibroin small-diameter vascular grafts via a novel electrospinning approach. J Biomed Mater Res A.

[51] Mostafavi F, Golshan Ebrahimi N (2012). Physical characterization and rheological behavior of polyurethane/poly (ϵ-caprolactone) blends, prepared by solution blending using dimethylacetamide. J Appl Polym Sci.

[52] Ansari M, Golzar M, Baghani M, Soleimani M (2018). Shape memory characterization of poly (ε-caprolactone)(PCL)/polyurethane (PU) in combined torsion-tension loading with potential applications in cardiovascular stent. Polym Test.

[53] Nguyen T-H (2013). A hybrid electrospun PU/PCL scaffold satisfied the requirements of blood vessel prosthesis in terms of mechanical properties, pore size, and biocompatibility. J Biomater Sci Polym Ed.

[54] Ren X (2015). Surface modification and endothelialization of biomaterials as potential scaffolds for vascular tissue engineering applications. Chem Soc Rev.

[55] Christenson EM (2004). Poly (carbonate urethane) and poly (ether urethane) biodegradation: in vivo studies. J Biomedi Mater Res Part A Off J Soc Biomater Jpn Soc Biomater Aust Soc Biomater Korean Soc Biomater.

[56] Xue L, Greisler HP (2003). Biomaterials in the development and future of vascular grafts. J Vasc Surg.

[57] Hashizume R (2013). The effect of polymer degradation time on functional outcomes of temporary elastic patch support in ischemic cardiomyopathy. Biomaterials.

[58] Sharifpoor S (2011). Functional characterization of human coronary artery smooth muscle cells under cyclic mechanical strain in a degradable polyurethane scaffold. Biomaterials.

[59] Ye S-H (2014). Nonthrombogenic, biodegradable elastomeric polyurethanes with variable sulfobetaine content. ACS Appl Mater Interfaces.

[60] Li Q, Mu L, Zhang F, Mo Z, Jin C, Qi W (2017). Manufacture and property research of heparin grafted electrospinning PCU artificial vascular scaffolds. Mater Sci Eng C.

[61] Gostev, A.A., et al., In vivo stability of polyurethane-based electrospun vascular grafts in terms of chemistry and mechanics. Polymers. 2020;12(4):845. 10.3390/polym12040845 PMC7240619 32272564

[62] Zhang H. Surface characterization techniques for polyurethane biomaterials, in Advances in polyurethane biomaterials. Amsterdam: Elsevier; 2016. p. 23–73.

[63] Liu S (2013). Bilayered vascular grafts based on silk proteins. Acta Biomater.

[64] Esmaeili S (2019). An artificial blood vessel fabricated by 3D printing for pharmaceutical application. Nanomed J.

[65] Mi H-Y (2018). Manipulating the structure and mechanical properties of thermoplastic polyurethane/polycaprolactone hybrid small diameter vascular scaffolds fabricated via electrospinning using an assembled rotating collector. J Mech Behav Biomed Mater.

[66] Jing X (2015). Electrospinning thermoplastic polyurethane/graphene oxide scaffolds for small diameter vascular graft applications. Mater Sci Eng C.

[67] Seifalian AM (2003). In vivo biostability of a poly (carbonate-urea) urethane graft. Biomaterials.

[68] Ahmed M, Hamilton G, Seifalian AM (2014). The performance of a small-calibre graft for vascular reconstructions in a senescent sheep model. Biomaterials.

[69] Zhang J, Doll BA, Beckman EJ, Hollinger JO (2003). A biodegradable polyurethane-ascorbic acid scaffold for bone tissue engineering. J Biomed Mater Res Part A Off J Soc Biomater Jpn Soc Biomater Aust Soc Biomater Korean Soc Biomater.

[70] Blit PH, Battiston KG, Yang M, Paul Santerre J, Woodhouse KA (2012). Electrospun elastin-like polypeptide enriched polyurethanes and their interactions with vascular smooth muscle cells. Acta Biomater.

[71] Huang C (2011). Electrospun collagen–chitosan–TPU nanofibrous scaffolds for tissue engineered tubular grafts. Colloids Surf B: Biointerfaces.

[72] Wang H (2012). Fabrication of PU/PEGMA crosslinked hybrid scaffolds by in situ UV photopolymerization favoring human endothelial cells growth for vascular tissue engineering. J Mater Sci Mater Med.

[73] Kucinska-Lipka J (2015). Fabrication of polyurethane and polyurethane based composite fibres by the electrospinning technique for soft tissue engineering of cardiovascular system. Mater Sci Eng C.

[74] Jia L (2013). Biocompatibility evaluation of protein-incorporated electrospun polyurethane-based scaffolds with smooth muscle cells for vascular tissue engineering. J Mater Sci.

[75] Wang H (2012). Co-electrospun blends of PU and PEG as potential biocompatible scaffolds for small-diameter vascular tissue engineering. Mater Sci Eng C.

[76] Oh B, Lee CH (2013). Nanofiber for cardiovascular tissue engineering. Expert Opin Drug Deliv.

[77] Khan M, Meduru S, Gogna R, Madan E, Citro L, Kuppusamy ML, Sayyid M, Mostafa M, Hamlin RL, Kuppusamy P (2012). Oxygen cycling in conjunction with stem cell transplantation induces NOS3 expression leading to attenuation of fibrosis and improved cardiac function. Cardiovasc Res.

[78] Li S, Sengupta D, Chien S (2014). Vascular tissue engineering: from in vitro to in situ. Wiley Interdiscip Rev Syst Biol Med.

[79] Gui L (2016). Implantable tissue-engineered blood vessels from human induced pluripotent stem cells. Biomaterials.

[80] Narazaki G, Uosaki H, Teranishi M, Okita K, Kim B, Matsuoka S, Yamanaka S, Yamashita JK (2008). Directed and systematic differentiation of cardiovascular cells from mouse induced pluripotent stem cells. Circulation.

[81] Liu Y (2003). Experimental study on constructing small-caliber artery by tissue engineering approach. Zhonghua wai ke za zhi [Chinese journal of surgery].

[82] Zhang WJ (2007). Tissue engineering of blood vessel. J Cell Mol Med.

[83] Shin’oka T (2005). Midterm clinical result of tissue-engineered vascular autografts seeded with autologous bone marrow cells. J Thorac Cardiovasc Surg.

[84] Chue W-L (2004). Dog peritoneal and pleural cavities as bioreactors to grow autologous vascular grafts. J Vasc Surg.

[85] L'heureux N (1998). A completely biological tissue-engineered human blood vessel. FASEB J.

[86] Roh JD (2010). Tissue-engineered vascular grafts transform into mature blood vessels via an inflammation-mediated process of vascular remodeling. Proc Natl Acad Sci.

[87] Huang NF, Li S (2008). Mesenchymal stem cells for vascular regeneration.

[88] Hilfiker A (2011). Mesenchymal stem cells and progenitor cells in connective tissue engineering and regenerative medicine: is there a future for transplantation?. Langenbeck's Arch Surg.

[89] Maul TM (2011). Mechanical stimuli differentially control stem cell behavior: morphology, proliferation, and differentiation. Biomech Model Mechanobiol.

[90] Park JS (2007). Mechanobiology of mesenchymal stem cells and their use in cardiovascular repair. Front Biosci.

[91] Campagnoli C (2001). Identification of mesenchymal stem/progenitor cells in human first-trimester fetal blood, liver, and bone marrow. Blood J Am Soc Hematol.

[92] Bieback K (2008). Comparing mesenchymal stromal cells from different human tissues: bone marrow, adipose tissue and umbilical cord blood. Biomed Mater Eng.

[93] Rebelatto C (2008). Dissimilar differentiation of mesenchymal stem cells from bone marrow, umbilical cord blood, and adipose tissue. Exp Biol Med.

[94] Gharaibeh B, Drowley L, Huard J. Muscle-derived stem cells: a model for stem cell therapy in regenerative medicine. Stem Cells Regenerative Medicine. Edited by: Appasani K, Appasani RK. New York: Humana Press; 2011. p. 565–578.

[95] Gharaibeh B, Drowley L, Huard J (2011). Muscle-derived stem cells: a model for stem cell therapy in regenerative medicine, in Stem Cells & Regenerative Medicine.

[96] Torsney E, Xu Q (2011). Resident vascular progenitor cells. J Mol Cell Cardiol.

[97] Caplan AI (2008). All MSCs are pericytes?. Cell Stem Cell.

[98] Medina RJ, Barber CL, Sabatier F, Dignat-George F, Melero-Martin JM, Khosrotehrani K, Ohneda O, Randi AM, Chan JKY, Yamaguchi T, van Hinsbergh VWM, Yoder MC, Stitt AW (2017). Endothelial progenitors: a consensus statement on nomenclature. Stem Cells Transl Med.

[99] Semenov OV (2010). Multipotent mesenchymal stem cells from human placenta: critical parameters for isolation and maintenance of stemness after isolation. Am J Obstet Gynecol.

[100] De Coppi P (2007). Isolation of amniotic stem cell lines with potential for therapy. Nat Biotechnol.

[101] Troyer DL, Weiss ML (2008). Concise review: Wharton's jelly-derived cells are a primitive stromal cell population. Stem Cells.

[102] Pu L (2017). Compared to the amniotic membrane, Wharton’s jelly may be a more suitable source of mesenchymal stem cells for cardiovascular tissue engineering and clinical regeneration. Stem Cell Res Ther.

[103] Weber B, Zeisberger S, Hoerstrup S (2011). Prenatally harvested cells for cardiovascular tissue engineering: fabrication of autologous implants prior to birth. Placenta.

[104] McCloskey KE, Gilroy ME, Nerem RM (2005). Use of embryonic stem cell-derived endothelial cells as a cell source to generate vessel structures in vitro. Tissue Eng.

[105] Gan S (2003). Tissue engineering of blood vessels with endothelial cells differentiated from mouse embryonic stem cells. Cell Res.

[106] Lopera Higuita M (2020). And L.G. Griffiths, *small diameter xenogeneic extracellular matrix scaffolds for vascular applications*. Tissue Eng B Rev.

[107] Petrović ZS (1991). The effect of crosslinking on properties of polyurethane elastomers. J Appl Polym Sci.

[108] Oprea S (2010). The effect of chain extenders structure on properties of new polyurethane elastomers. Polym Bull.

[109] Karchin A, Simonovsky FI, Ratner BD, Sanders JE (2011). Melt electrospinning of biodegradable polyurethane scaffolds. Acta Biomater.

[110] Melchiorri A (2015). Contrasting biofunctionalization strategies for the enhanced endothelialization of biodegradable vascular grafts. Biomacromolecules.

[111] Safikhani MM (2017). Bi-layered electrospun nanofibrous polyurethane-gelatin scaffold with targeted heparin release profiles for tissue engineering applications. J Polym Eng.

[112] Hao H (2016). Synthesis and characterization of biodegradable lysine-based waterborne polyurethane for soft tissue engineering applications. Biomater Sci.

[113] Segan S, Jakobi M, Khokhani P, Klimosch S, Billing F, Schneider M, Martin D, Metzger U, Biesemeier A, Xiong X, Mukherjee A, Steuer H, Keller BM, Joos T, Schmolz M, Rothbauer U, Hartmann H, Burkhardt C, Lorenz G, Schneiderhan-Marra N, Shipp C (2020). Systematic investigation of polyurethane biomaterial surface roughness on human immune responses in vitro. Biomed Res Int.

[114] Xu L-C, Meyerhoff ME, Siedlecki CA (2019). Blood coagulation response and bacterial adhesion to biomimetic polyurethane biomaterials prepared with surface texturing and nitric oxide release. Acta Biomater.

[115] Myrna KE (2012). Substratum topography modulates corneal fibroblast to myofibroblast transformation. Invest Ophthalmol Vis Sci.

[116] Kilic C (2014). A collagen-based corneal stroma substitute with micro-designed architecture. Biomater Sci.

[117] Le Saux G (2011). The relative importance of topography and RGD ligand density for endothelial cell adhesion. PLoS One.

[118] Liang R (2018). Macrophage polarization in response to varying pore sizes of 3D polyurethane scaffolds. J Biomed Nanotechnol.

[119] Xu L-C, Siedlecki CA (2012). Submicron-textured biomaterial surface reduces staphylococcal bacterial adhesion and biofilm formation. Acta Biomater.

[120] Andorko JI, Jewell CM (2017). Designing biomaterials with immunomodulatory properties for tissue engineering and regenerative medicine. Bioeng Transl Med.

[121] Morita Y, Sakamoto H, Suye S-i (2017). Characterization of protein adsorption on stretched polyurethane nanofibers prepared by electrospinning. RSC Adv.

[122] Du B (2020). A waterborne polyurethane 3D scaffold containing PLGA with a controllable degradation rate and an anti-inflammatory effect for potential applications in neural tissue repair. J Mater Chem B.

[123] Liu T-M, Wu X-Z, Qiu Y-R (2016). Enhanced biocompatibility and antibacterial property of polyurethane materials modified with citric acid and chitosan. J Biomater Sci Polym Ed.

[124] Gorna K, Gogolewski S (2003). Preparation, degradation, and calcification of biodegradable polyurethane foams for bone graft substitutes. J Biomed Mater Res Part A Off J Soc Biomater Jpn Soc Biomater Aust Soc Biomater Korean Soc Biomater.

[125] Anderson JM, Hiltner A, Wiggins MJ, Schubert MA, Collier TO, Kao WJ (1998). Recent advances in biomedical polyurethane biostability and biodegradation. Polym Int..

[126] Szycher M (1988). Biostability of polyurethane elastomers: a critical review. J Biomater Appl.

[127] Ghista DN, Reul H (1977). Optimal prosthetic aortic leaflet valve: design parametric and longevity analyses: development of the Avcothane-51 leaflet valve based on the optimum design analysis. J Biomech.

[128] Xie F, Zhang T, Bryant P, Kurusingal V, Colwell JM, Laycock B (2019). Degradation and stabilization of polyurethane elastomers. Prog Polym Sci.

[129] Hsu S-h, Lin Z-C (2004). Biocompatibility and biostability of a series of poly (carbonate) urethanes. Colloids Surf B: Biointerfaces.

[130] Clemitson I. Castable polyurethane elastomers. Boca Raton: CRC Press; 2015.

[131] Khan I (2005). Analysis and evaluation of a biomedical polycarbonate urethane tested in an in vitro study and an ovine arthroplasty model. Part I: materials selection and evaluation. Biomaterials.

[132] Singh C, Wang X (2017). Metal ion-loaded nanofibre matrices for calcification inhibition in polyurethane implants. J Funct Biomater.

[133] Shimada K (2017). The effect of a silk fibroin/polyurethane blend patch on rat vessels. Organogenesis.

[134] Thomas V, Jayabalan M (2009). A new generation of high flex life polyurethane urea for polymer heart valve—studies on in vivo biocompatibility and biodurability. J Biomed Mater Res Part A Off J Soc Biomater Jpn Soc Biomater Aust Soc Biomater Korean Soc Biomater.

[135] Amado JCQ (2020). Evaluation of elastomeric heat shielding materials as insulators for solid propellant rocket motors: a short review. Open Chem.

[136] Slater C, Davis C, Strangwood M (2011). Compression set of thermoplastic polyurethane under different thermal–mechanical-moisture conditions. Polym Degrad Stab.

[137] Pretsch T, Jakob I, Müller W (2009). Hydrolytic degradation and functional stability of a segmented shape memory poly (ester urethane). Polym Degrad Stab.

[138] Stevenson J, Kusy R (1995). Structural degradation of polyurethane-based elastomeric modules. J Mater Sci Mater Med.

[139] Macocinschi D (2015). Thermal and hydrolytic stability of silver nanoparticle polyurethane biocomposites for medical applications. Polym Degrad Stab.

[140] Brown DW, Lowry RE, Smith LE (1980). Kinetics of hydrolytic aging of polyester urethane elastomers. Macromolecules.

[141] Cauich-Rodríguez JV, et al. Degradation of polyurethanes for cardiovascular applications. Adv Biomater Sci Biomed Appl. 2013:51–82.

[142] Schollenberger C, Stewart F (1973). Thermoplastic polyurethane hydrolysis stability. Die Angewandte Makromolekulare Chemie Appl Macromol Chem Phys.

[143] Gunatillake PA (1992). Polyurethane elastomers based on novel polyether macrodiols and MDI: synthesis, mechanical properties, and resistance to hydrolysis and oxidation. J Appl Polym Sci.

[144] Zdrahala RJ, Zdrahala IJ (1999). Biomedical applications of polyurethanes: a review of past promises, present realities, and a vibrant future. J Biomater Appl.

[145] Skarja G, Woodhouse K (2000). Structure-property relationships of degradable polyurethane elastomers containing an amino acid-based chain extender. J Appl Polym Sci.

[146] Loh XJ, Goh SH, Li J (2007). Hydrolytic degradation and protein release studies of thermogelling polyurethane copolymers consisting of poly [(R)-3-hydroxybutyrate], poly (ethylene glycol), and poly (propylene glycol). Biomaterials.

[147] Simmons A, Hyvarinen J, Poole-Warren L (2006). The effect of sterilisation on a poly (dimethylsiloxane)/poly (hexamethylene oxide) mixed macrodiol-based polyurethane elastomer. Biomaterials.

[148] Ward R, Jones R. Polyurethanes and silicone polyurethane copolymers. In: Ducheyne P, editor. Comprehensive Biomaterials II. 2nd ed. Boca Raton: O.E. Ltd; 2017.

[149] Pilichowski JF, Liptaj T, Morel M, Terriac E, Baba M (2003). Cross-linking of polybutadiene: correlation between solid-state 1H NMR spectroscopy, thermoporosimetry, densimetry and crystallinity measurements. Polym Int.

[150] Mondal S, Martin D (2012). Hydrolytic degradation of segmented polyurethane copolymers for biomedical applications. Polym Degrad Stab.

[151] Hong Park S (2009). Mechanical and surface properties and hydrolytic stability of cycloaliphatic polyester-based waterborne polyurethanes modified with fluoro oligomer. J Appl Polym Sci.

[152] Kim YD, Kim SC (1998). Effect of chemical structure on the biodegradation of polyurethanes under composting conditions. Polym Degrad Stab.

[153] Wolfram N, et al. Stabilization of polyesters with polycarbodiimide. Google Patents No: US3193523A; 1965.

[154] Loew F. Elastomeric thermoplastic polyester polyurethane compositions stabilized against hydrolysis. Google Patents No: US3716502A; 1973.

[155] Labow R, Meek E, Santerre J (2001). Hydrolytic degradation of poly (carbonate)-urethanes by monocyte-derived macrophages. Biomaterials.

[156] Labow RS, Meek E, Santerre JP (1999). The biodegradation of poly (urethane) s by the esterolytic activity of serine proteases and oxidative enzyme systems. J Biomater Sci Polym Ed.

[157] Santerre J (2005). Understanding the biodegradation of polyurethanes: from classical implants to tissue engineering materials. Biomaterials.

[158] Takahara A, et al. Effect of polyol chemistry on the in vitro biostability of segmented polyurethanes, in Artificial Heart. Tokyo: Springer; 1991. p. 77–83.

[159] Takahara A, Hergenrother RW, Coury AJ, Cooper SL (1992). Effect of soft segment chemistry on the biostability of segmented polyurethanes. II. In vitro hydrolytic degradation and lipod sorption. J Biomed Mater Res.

[160] Christenson E, Anderson J, Hiltner A (2007). Biodegradation mechanisms of polyurethane elastomers. Corros Eng Sci Technol.

[161] Zhao Q, Topham N, Anderson JM, Hiltner A, Lodoen G, Payet CR (1991). Foreign-body giant cells and polyurethane biostability: in vivo correlation of cell adhesion and surface cracking. J Biomed Mater Res.

[162] Anderson JM (1993). Mechanisms of inflammation and infection with implanted devices. Cardiovasc Pathol.

[163] Schubert MA (1995). Oxidative biodegradation mechanisms of biaxially strained poly (etherurethane urea) elastomers. J Biomed Mater Res.

[164] Mathur AB, Collier TO, Kao WJ, Wiggins M, Schubert MA, Hiltner A (1997). In vivo biocompatibility and biostability of modified polyurethanes. J Biomed Mater Res..

[165] Martin DJ, Poole Warren LA, Gunatillake PA, McCarthy SJ, Meijs GF, Schindhelm K (2000). Polydimethylsiloxane/polyether-mixed macrodiol-based polyurethane elastomers: biostability. Biomaterials.

[166] Christenson EM, Anderson JM, Hiltner A (2006). Antioxidant inhibition of poly (carbonate urethane) in vivo biodegradation. J Biomed Mater Res Part A Off J Soc Biomater Jpn Soc Biomater Aust Soc Biomater Korean Soc Biomateri.

[167] Wu Y, Sellitti C, Anderson JM, Hiltner A, Lodoen GA, Payet CR (1992). An FTIR–ATR investigation of in vivo poly (ether urethane) degradation. J Appl Polym Sci.

[168] Schubert MA (1997). Comparison of two antioxidants for poly (etherurethane urea) in an accelerated in vitro biodegradation system. J Biomed Mater Res Off J Soc Biomater Jpn Soc Biomater.

[169] Simmons A (2004). Long-term in vivo biostability of poly (dimethylsiloxane)/poly (hexamethylene oxide) mixed macrodiol-based polyurethane elastomers. Biomaterials.

[170] Christenson EM, Anderson JM, Hiltner A (2004). Oxidative mechanisms of poly (carbonate urethane) and poly (ether urethane) biodegradation: in vivo and in vitro correlations. J Biomedi Mater Res Part A Off J Soc Biomater Jpn Soc Biomater Aust Soc Biomater Korean Soc Biomater.

[171] Scott G (1995). Initiation processes in polymer degradation. Polym Degrad Stab.

[172] Hawkins WL (1984). Polymer degradation, in Polymer Degradation and Stabilization.

[173] Hainsworth S (2007). An environmental scanning electron microscopy investigation of fatigue crack initiation and propagation in elastomers. Polym Test.

[174] Rabek JF. Physical aspects of the photodegradation of polymers, in Polymer Photodegradation. London: Springer; 1995. p. 1–23.

[175] Papadopoulos I, Thomas A, Busfield J (2008). Rate transitions in the fatigue crack growth of elastomers. J Appl Polym Sci.

[176] Cho K, Jang WJ, Lee D, Chun H, Chang YW (2000). Fatigue crack growth of elastomers in the swollen state. Polymer.

[177] Hess F (1992). Development and long-term fate of a cellular lining in fibrous polyurethane vascular prostheses implanted in the dog carotid and femoral artery. A scanning and light microscopical study up to 53 months after implantation. J Cardiovasc Surg.

[178] Santerre J (1994). Biodegradation evaluation of polyether and polyester-urethanes with oxidative and hydrolytic enzymes. J Biomed Mater Res.

[179] Glickman M (2001). Multicenter evaluation of a polytetrafluoroethylene vascular access graft as compared with the e-PTFE vascular access graft in hemodialysis applications. J Vasc Surg.

[180] Vakili H, et al. Enhanced hemocompatibility of a PEGilated polycarbonate based segmented polyurethane. Int J Polym Mater Polym Biomater. 2020:1–9. 10.1080/00914037.2020.1857760.

